# Warming and Nitrogen Addition Change the Soil and Soil Microbial Biomass C:N:P Stoichiometry of a Meadow Steppe

**DOI:** 10.3390/ijerph16152705

**Published:** 2019-07-29

**Authors:** Shiwei Gong, Tao Zhang, Jixun Guo

**Affiliations:** 1 College of Resources and Environment, Shanxi University of Finance & Economics, Taiyuan 030006, China; 2 Institute of Grassland Sciences, Northeast Normal University, Key Laboratory for Vegetation Ecology, Ministry of Education, Changchun 130024, China

**Keywords:** warming, N addition, soil C:N:P stoichiometry, soil microbial biomass C:N:P stoichiometry, Meadow steppe

## Abstract

Soil and soil microbial biomass (SMB) carbon: nitrogen: phosphorus (C:N:P) stoichiometry are important parameters to determine soil balance of nutrients and circulation of materials, but how soil and SMB C:N:P stoichiometry is affected by climate change remains unclear. Field experiments with warming and N addition had been implemented since April 2007. Infrared radiators were used to manipulate temperature, and aqueous ammonium nitrate (10 g m^−2^ yr^−1^) was added to simulate nitrogen deposition. We found that molar nutrient ratios in the soil averaged 60:11:1, warming and warming plus N addition reduced soil C:N by 14.1% and 20% (*P* < 0.01), and reduced soil C:P ratios by 14.5% and 14.8% (*P* < 0.01). N addition reduced soil C:N significantly by 17.6% (*P* < 0.001) (Figure 2B,D). N addition and warming plus N addition increased soil N:P significantly by 24.6% and 7.7% (*P* < 0.01). The SMB C:N, C:P and N:P ratios increased significantly with warming, N addition and warming plus N addition. Warming and N addition increased the correlations between SOC and soil microbial biomass C (SMBC), soil total P and soil microbial biomass P (SMBP), warming increased the correlation between the soil total N and soil microbial biomass N (SMBN). After four years’ treatment, our results demonstrated that the combined effects of warming and N fertilization could change the C, N, P cycling by affecting soil and SMB C:N:P ratios significantly and differently. At the same time, our results suggested SMB might have weak homeostasis in Sonnen Grassland and warming and N addition would ease N-limitation but aggravate P-limitation in northeastern China. Furthermore, these results further the current demonstration of the relationships between the soil and SMB C:N:P stoichiometry in response to global change in temperate grassland ecosystems.

## 1. Introduction

Terrestrial ecosystems have been influenced by human-induced environmental changes including nitrogen (N) deposition and global warming [1]. The global change may profoundly affect biogeochemical interactions among key elements such as carbon (C), N, and phosphorus (P). Warming can directly change soil temperature and moisture [2]. N deposition caused by human activities is 30% higher than that from natural terrestrial input [3]. Warming and N deposition might affect ecosystem stoichiometry to change the structure and function of the ecosystem.

Soil C:N:P stoichiometry is considered to be an important indicator of nutrient status during soil development [4], and the availability and limitation of essential nutrients can feed back on soil organic C (SOC) dynamics [5]. SMB plays a vital role in soil nutrient transformations. Soil microorganisms control the decomposition of soil organic matter, thereby affecting the C, N and P balance of terrestrial ecosystems, and nutrient availability for plants [6,7]. Soil stoichiometry can strongly influence the organisms’ C: N: P stoichiometry [8], therefore, the relationship between soil and SMB C:N:P ratio is important to understand microbial nutrient limitations in soils [9,10].

Currently, researchers apply to plant, litter and soil C:N:P stoichiometry in biogeochemical cycles, ecosystem stability and other fields [4,11,12]. Some research reported that N fertilization reduced the C:N and increased the N:P of the soil, plants [13,14], and freshwater ecosystems [15]. Han et al. reported an N: P of 15.3 in grass leaves, based on a study of 213 plant species in China [16]. To date, a lot of research has been done on plants and aquatic ecosystems, but less attention has been paid to terrestrial ecosystems. Our understanding of C:N:P ratios in soil and SMB is relatively limited. Warming and N fertilization affect the stoichiometric of meadow steppe in response to global climate change also remain unclear. Consequently, our objective was to assess how global climate change affected soil and SMB elemental ratios.

Sonnen Grassland lies in the eastern of Eurasian grassland, which is the most typical meadow steppe. The average temperature of Sonnen Grassland elevated 2 °C in the last two decades [17], and average atmospheric N deposition is approximately 10.5 g m^−2^ yr^−1^ [18]. To understand the influence of global warming and N addition on soil and SMB C:N:P stoichiometry, we conducted an artificial warming and N addition experiment in Northeast China. The objectives of this study included: (1) To what extent do warming and N addition affect soil and SMB C:N:P stoichiometry, (2) whether or not there are interactive effects between warming and N addition on soil and SMB C:N:P stoichiometry, (3) whether or not warming and N addition affect the correlation between soil and SMB C:N:P stoichiometry. 

## 2. Materials and Methods

### 2.1. Study Site

**Figure 1:**
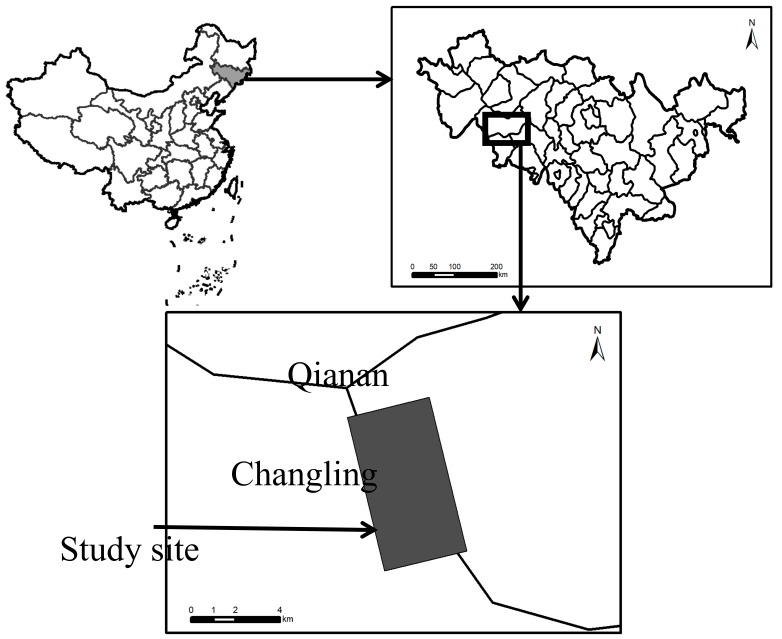
Location of the study site.

Four-year experiments were conducted in the Sonnen Grassland of northeast China (123°44′ E and 44°40′ N) (Figure 1). The mean annual temperature is 6.4 °C. The mean annual rainfall is 470 mm, which occurs between June and August [19]. The vegetation is dominated by the perennial grass *Leymus chinensis* (Trin.) Travel. and *Phragmites communis*, accompanying vegetation are *Carex duriuscula* C. A. Mey., *Rhizoma phragmites*, and *Kalimeris integrifolia* Turcz. Ex DC. Chernozem is the main soil type with 2.0% of soil organic carbon content, 1.4% of soil humus, 0.15% of total N and pH 8.14 ± 0.2 [19]. Carbonates don’t exist in the soil. 

### 2.2. Experimental Design

In the experiment, warming and N addition were fixed factors. The size of each plot was 2 × 3 m. There were four treatments: Control (C), warming (W), N addition (N), and warming plus N addition (WN). Infrared radiators (Kalglo Electronics Inc. Bethlehem, PA, MSR-2420, USA) were used to manipulate temperature, in each control and N addition plot, a ‘dummy’ heater with the same shape and size was installed to simulate the shading effects of the infrared radiator. 

A pulse of aqueous ammonium nitrate (10 g m^−2^ yr^−1^) was added to simulate nitrogen deposition on the first day of May each year. The same amount of water (equivalent to ~2 mm of rainfall) was applied to N addition and ambient N plots (i.e., without N addition).

### 2.3. Soil Microclimate

An ECH_2_O Dielectric Aquameter (EM50/R Decagon Ltd., Pullman, WA, USA) was used to measure soil temperature and water content. For each subplot, soil temperature and water content (0–15 cm) were measured at 08:00–09:00 a.m. in May, June, July, August, September and October from 2008 to 2010. 

### 2.4. Soil Sampling

Soil samples (0–15 cm) were collected once a month from all the plots from May to October in 2008, 2009 and 2010. Samples were collected with a cylindrical soil sampler (5-cm inner diameter, 15-cm length) in the 0–15 cm layer from three random locations in each plot to account for soil heterogeneity, then sieved using a 2 mm-diameter soil screen to remove roots, gravel, rocks and stones. One portion of samples were immediately used to measure SMB C, N, P, and then stored at 4 °C. Another portion was air-dried before conducting chemical analysis.

### 2.5. SOC, and Soil N and P

SOC was measured with the dichromate oxidation method [20]. Soil total N was measured with the Kjeldahl method [21]. Soil total P was firstly digested in sulfuric acid, then subsequently quantified with ICP Elemental Analyzer (Bruker Analysis Instrument Ltd., Karlsruhe, BW Germany). 

### 2.6. Soil Microbial Biomass C, N, P

Chloroform fumigation-extraction (CFE) technique was used to measure SMB C, N and P [22,23,24]: 10 g d.w. equivalent of soil was fumigated for 24 h at 25 °C and extracted with 0.5 M K_2_SO_4_ (for C and N) or 0.5 M NaHCO_3_ (for P). And correction for soil P sorption was done in the SMB-P determination. C, N and P of unfumigated soils were measured in the same way. SMB element content was calculated as the difference between the fumigated and unfumigated samples. 

### 2.7. Statistical Analysis

Assessing the temporal variation and the effects of warming and N addition on soil and SMB C, N, P and C:N:P stoichiometry by using repeated measures ANOVAs. Warming, N addition, and warming plus N addition were treated as between-subject factors. Determine the relationships between soil and SMB C, N, P and C:N:P stoichiometry and soil temperature, soil water content by using linear regression analyses. Significance level at our statistical analysis is a = 0.05. Statistical analyses used SPSS (SPSS Institute Inc., Chicago, IL, USA). Data are reported as mean ±SE.

## 3. Results

### 3.1. Soil Microclimate

**Figure 2:**
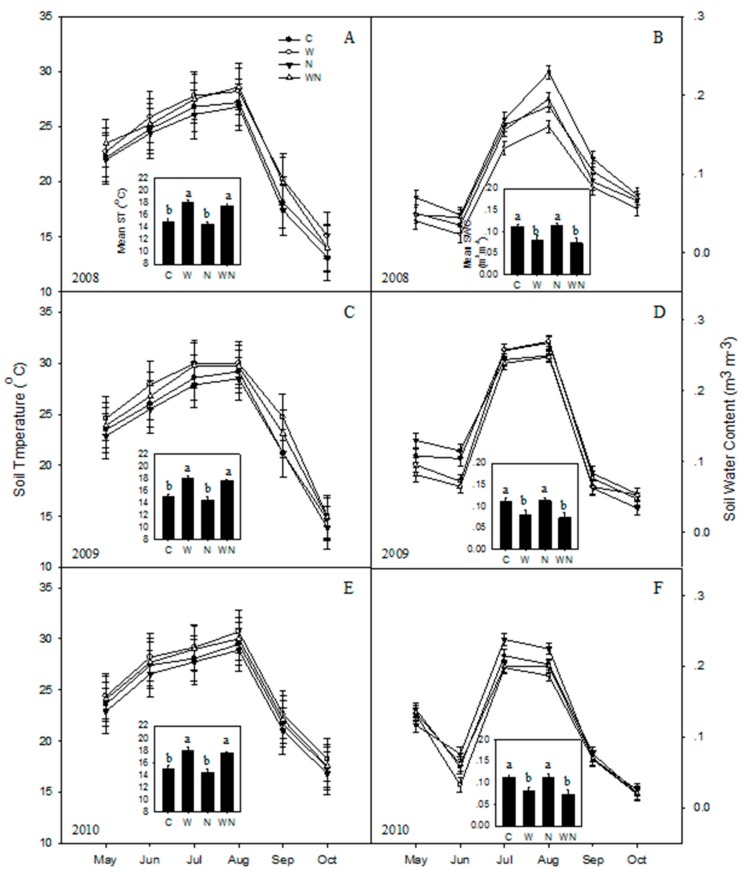
Seasonal variations of soil temperature (**A**: 2008, **C**: 2009, **E**: 2010) and water content (**B**: 2008, **D**: 2009, **F**: 2010) in response to warming and N addition. C: Control; W: Warming; N: N addition; WN: Warming plus N addition. Vertical bars indicate standard error of the mean (n = 6). Different lowercase letters indicate significant differences (*P* < 0.05).

Soil temperature showed a seasonal response from 2008 to 2010. Each year, the soil temperature exhibited a unimodal peak in August (Figure 2A,C,E). The soil water content showed a seasonal trend and exhibited peaks in July and August (Figure 2B,D,F). Warming and warming plus N increased soil temperature 1.1 °C (*P* < 0.05), but reduced soil water content (*P* < 0.05). 

### 3.2. SOC, Soil Total N, total P 

**Figure 3:**
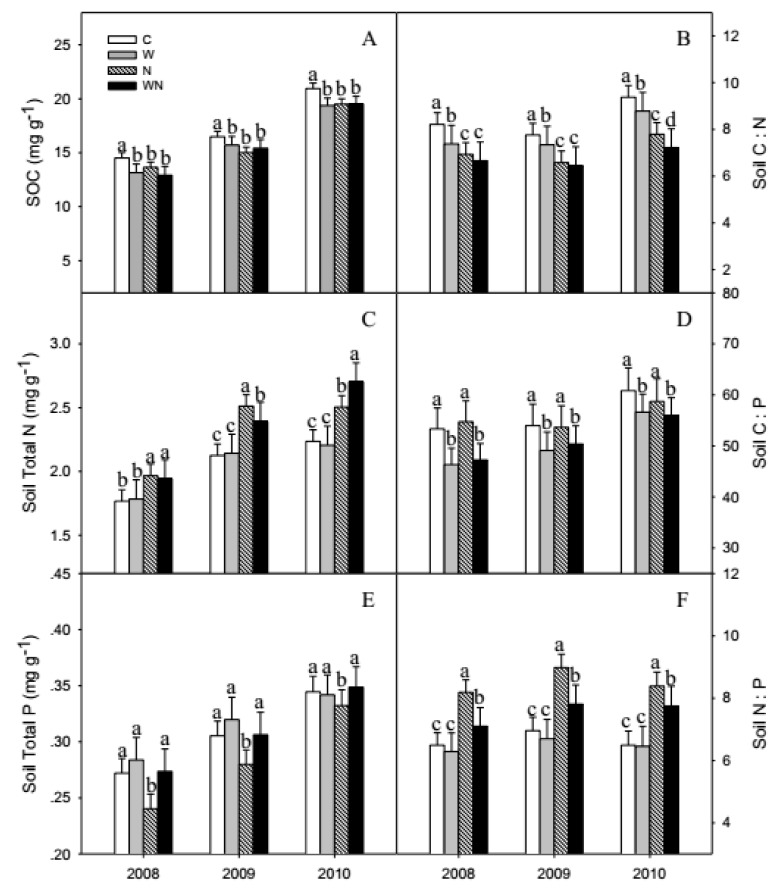
Responses of SOC (**A**), soil total N (**C**), total P (**E**) contents and C:N (**B**), C:P (**D**), N:P (**F**) stoichiometry to warming and N addition. Vertical bars represent the standard error of the mean (n = 6). Different lowercase letters indicate significant differences (*P* < 0.05). See Figure 2 for abbreviations.

SOC, soil total N and total P showed increasing trends from 2008 to 2010 and increased by 40%, 25% and 27.3%, respectively. Warming and N addition significantly (*P* < 0.001) reduced the SOC by 7.2% and 7.3% on average, respectively (Figure 3A). N addition and warming plus N addition increased the soil total N by 17.3% (*P* < 0.001) and 15.7% (*P* < 0.01), respectively. N addition significantly reduced soil total P by 14.9% (*P* < 0.01) (Figure 3E). 

The soil C:N:P ratios averaged 60:11:1, warming and warming plus N addition reduced soil C:N by 14.1% and 20% (*P* < 0.01), and reduced soil C:P ratios by 14.5% and 14.8% (*P* < 0.01). N addition reduced soil C:N significantly by 17.6% (*P* < 0.001) (Figure 3B,D). N addition and warming plus N addition increased soil N:P significantly by 24.6% and 7.7% (*P* < 0.01) (Figure 3F).

### 3.3. Soil Microbial Biomass C, N, P

**Figure 4:**
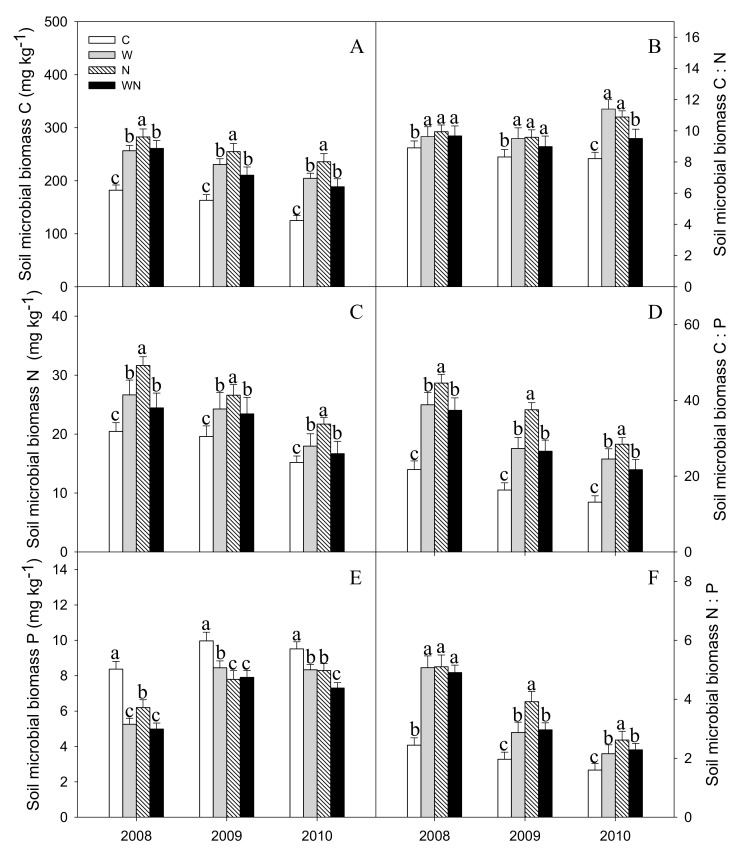
Responses of soil microbial biomass C (**A**), N (**C**), P(**E**) contents and C:N (**B**), C:P (**D**), N:P (**F**) stoichiometry to warming and N addition. Vertical bars represent the standard error of the mean (n = 6). Different lowercase letters indicate significant differences (*P* < 0.05). See Figure 2 for abbreviations.

Warming, N addition and warming plus N addition increased the SMBC content by 47%, 65% and 34%, respectively (*P* < 0.001) (Figure 4A). Warming and N addition increased the SMBN by 25% and 45%, respectively (*P* < 0.001) (Figure 4C). Warming, N addition and warming plus N addition reduced the SMBP by 21%, 24% and 27%, respectively (*P* < 0.01) (Figure 4E). 

The SMB C:N, C:P and N:P ratios increased significantly with warming, N addition and warming plus N addition (*P* < 0.01) (Figure 4B,D,F).

### 3.4. Correlation of Soil and Soil Microbial Biomass C, N, P With Soil Microclimate 

Correlation analysis showed that the correlations of soil and SMB C:N:P with soil microclimate were different in four treatments (Table 1).

SOC, soil total N, total P contents had positive correlation with soil temperature or water content, and the correlation of soil total P was the highest. Soil C:N:P had a positive correlation with soil temperature or water content, and soil C:P with soil temperature was the highest, soil N:P with soil water content was the highest. SMBC, SMBN, SMBP positively correlated with soil temperature or water content, but the correlation of SMBC was the lowest. Correlations between SMB C:N:P and soil temperature or water content were not evident (Table 1). 

Warming increased the correlations of soil total P with soil temperature but reduced the correlations of SOC, soil total N, total P and soil C:N:P with soil water content. N addition increased the correlations of soil C:N:P with soil water content but reduced the correlations with soil temperature (Table 1). Warming increased the correlations of SMBC and SMBN with soil temperature and water content and increased the correlations of SMB C:P and SMB N:P with soil temperature but reduced the correlations of SMBP with soil temperature and water content. N addition increased the correlations of SMBC, SMB C:P and SMB N:P with soil temperature and water content but reduced the correlations of SMBP with soil temperature and water content (Table 1).

### 3.5. Correlation of SOC, Soil Total N, Total P With Soil Microbial Biomass C, N, P

**Figure 5:**
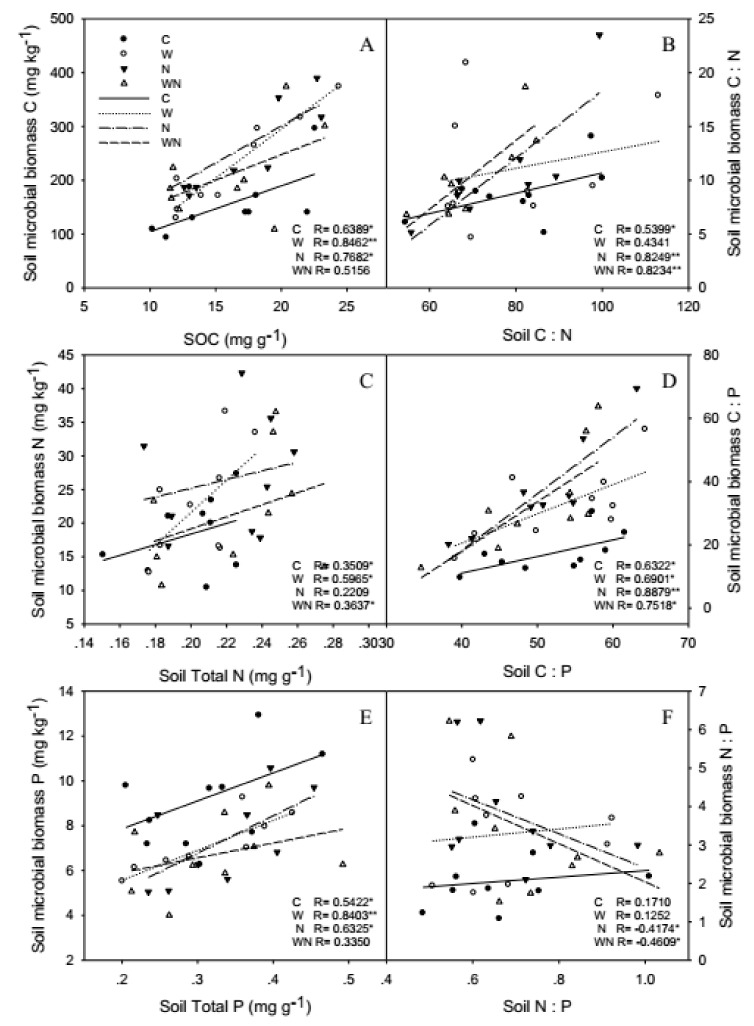
Responses of correlation of soil and soil microbial biomass C (**A**), N (**C**), P(**E**) contents and C:N (**B**), C:P (**D**), N:P (F) stoichiometry to warming and N addition Vertical bars represent the standard error of the mean (*n* = 6). See Figure 2 for abbreviations.

SOC and SMBC, soil total P and SMBP had a positive correlation, but the correlation between soil total N and SMBN was weaker (Figure 5A,C,E). Soil and SMB C:N and C:P also had a positive correlation, but the correlation of N:P was not observed (Figure 5B,D,F).

Warming and N addition increased the correlations of SOC and SMBC, soil total P and SMBP, and warming increased the correlation of soil total N and SMBN (Figure 5A,C,E). Furthermore, N addition and warming plus N addition increased the correlations of soil and SMB C:N and C:P, but N:P had negative correlation (Figure 5B,D,F).

## 4. Discussion

### 4.1. Responses of Soil C: N: P Stoichiometry to Warming and N Addition 

In this study, warming reduced SOC, the soil C:N and C:P, some research has got the same results, and they also found warming increased soil N:P [25,26], it did not occur in our studies. Some researchers have reported that warming decreased SOC in grasslands [27] and increased soil total N [28], because warming promotes the growth of plants and microorganisms, and promotes soil enzymes activity, which is conducive to the decomposition and fixation of organic compounds by microorganisms and soil enzymes, and is conducive to the absorption of C by plants, a large amount of C flows into plants and microorganisms, so warming reduced SOC. In our results, warming did not affect soil total N, might because warming increases the aboveground biomass and increases the capacity of plant N uptake [29,30], so soil total N did not increase. Treatment effects on soil C:N, C:P and N:P were caused by simultaneous changes in SOC, soil total N and total P content. Warming and warming plus N addition reduced soil C:N and C:P, because high soil water content increases the diffusivity of P, enhancing the uptake by plants and microorganism and decreased the availability of N [31], but warming reduced the soil water content, which increased the availability of N and reduced the diffusivity of P, and warming and warming plus N addition reduced the relationship between SOC, soil total P and soil water content. Effect of warming on soil N:P may be negligible, indicating that soils may have high stoichiometric homeostasis under global warming.

In the Sonnen Grassland, N addition reduced SOC, soil total P and soil C:N, but increased soil total N and N:P, which were same with some studies [25,32,33], but had no effect with soil C:P, which because N addition reduced SOC and soil total P content at the same time. Treatment effects on soil C:N, C:P and N:P ratios were caused by simultaneous changes in SOC, soil total N and total P contents. N addition enhances SMB and increases soil enzyme activity that promotes the mineralization of organic matter [34]. Meanwhile, excessive N and organic matter reduces soil C:N and accelerates the decomposition of organic matter and the release of nutrients [35,36]. Some studies show that N addition accelerated the N mineralization rate, and the additional nitrogen was absorbed by soil organic matter, reducing SOC and increasing the nitrogen release [37,38,39]. This viewpoint supported our results. N addition promoted microbes activity and increased the utilization of soil total P, thereby decreasing soil total P significantly [34,40]. Moreover, N fertilization promoted plant growth could stimulate P uptake [40,41]. The variations of C and N contents are large in the soil, but the low soil total P always led to high C:P and N:P [4]. 

### 4.2. Responses of Soil Microbial Biomass C: N: P Stoichiometry to Warming and N Addition 

The level of SMB C, N and P content had no significant change over the three growing seasons. Warming, N addition and warming plus N addition increased SMBC and SMBN contents, SMB C:N, C:P and N:P, but reduced SMBP content. Dijkstra et al. found warming reduced both the SMB P:C and SMBP [11], it just like our results. Warming and N addition, are conducive to the growth of plants and other organisms, promote the discharge of plant root exudates, promote the litter decomposition and soil microbial activity [42,43], so increase SMBC and SMBN contents. At same time, N addition increased soil N content, which is conducive to immobilization and absorption of nitrogen by microorganisms, finally increase soil microbial activity and the microbial absorption rate of nutrients, also increased SMBC and SMBN contents [44,45], and it also confirmed that the positive correlation of SMBC and SMBN with soil temperature and water content [46]. N addition reduced SMBP content because N addition increased soil N content and N availability, which caused microbial P-limitation [40,47]. 

### 4.3. Responses of the Correlation of Soil and Soil Microbial Biomass C: N: P Stoichiometry to Warming and N Addition 

Soil and SMB C, N, P were strongly and linearly related because the chemical composition of microbes (C, N, P content) was changed with the nutrient of the environment [48]. In our study, SOC and SMBC, soil total P and SMBP had a positive correlation, soil and SMB C:N and C:P also had a positive correlation, but the correlation of N:P was not observed (Figure 5B,D,F). Some studies have obtained the same results [49,50]. The SMB C:N and C:P are variable with soil stoichiometry, which might mean the SMB existences weaker homeostasis [51], so SMB was not a homeostatic system, and N and P are limiting factors for biological productivity in Sonnen Grassland. Nevertheless, the SMB C:N and C:P is expected to be highly variable because SMBN and SMBP turn over faster than SMBC [52].

Warming and N addition promote the growth of microorganisms, a lot of C and N in the soil are absorbed. Since warming and N addition reduced SOC but increased SMBC, which means warming and N addition promote the absorption of SOC by SMB, the lack of SOC is not conducive to SMBC, SOC with SMBC had positive correlation, so higher correlations between soil and SMBC, worse immobilization and transformation of microorganisms with C. But N and P are limiting factors in the soils of Sonnen Grassland, warming and N addition increased soil total N and SMBN, reduced soil total P and SMBP, and soil total N with SMBN and soil total P with SMBP had positive correlation, so higher correlations between soil and SMB N and P, better immobilization and transformation of microorganisms with N, but worse immobilization and transformation with P.

Soil and SMB N:P had a negative correlation in N addition and warming plus N addition treatments, this finding indicates that N addition and warming plus N addition increased soil N and caused soil P limitation, which promoted immobilization and transformation of soil microorganisms with N but inhibited immobilization and transformation with P.

## 5. Conclusions

In Sonnen Grassland of China, soil and SMB C:N:P stoichiometry were 60:11:1 and 58:5:1, respectively. This result was remarkably close to the C:N:P stoichiometry (61:5.3:1) for the soils of China [6] and to the global average SMB C:N:P stoichiometry (60:7:1) [53]. Warming and N addition increased the correlations of SOC and SMBC, soil total P and SMBP, and warming increased the correlation of soil total N and SMBN. In our study, the combined effects of warming and N addition could change the C, N, P cycling of the entire ecosystems by affecting soil and SMB C:N:P stoichiometry significantly and differently. It also suggested SMB might have weak homeostasis in Sonnen Grassland, and warming and N addition will ease N-limitation but aggravate P-limitation in northeastern China. Furthermore, these results further the current demonstration of the relationships between the soil and SMB C:N:P stoichiometry in response to global change in temperate grassland ecosystems.

## Figures and Tables

**Table 1:** Correlations between soil and SMB C, N, P and soil temperature as well as water content.

	Soil Temperature				Soil Water Content			
	C	W	N	WN	C	W	N	WN
SOC	0.429 *	0.371	0.486 *	0.483 *	0.681 *	0.141	0.733 **	0.174
Soil total N	0.467 *	0.475 *	0.332	0.483 *	0.685 *	0.429 *	0.369	0.422 *
Soil total P	0.569 *	0.733 **	0.543 *	0.781 **	0.867 **	0.371	0.876 **	0.365
Soil C:N	0.583 *	0.316	0.251	0.429 *	0.321	0.416 *	0.522 *	0.681 *
Soil C:P	0.691 *	0.439 *	0.468 *	0.367	0.677 *	0.333	0.989 **	0.201
Soil N:P	0.451 *	0.544 *	0.395	0.338	0.785 *	0.314	0.986 **	0.203
MBC	0.567 *	0.815 **	0.727 *	0.698 *	0.593 *	0.642 *	0.802 *	0.783 *
MBN	0.929 **	0.961 **	0.831 **	0.929 **	0.713 *	0.751 *	0.839 **	0.852 **
MBP	0.891 *	0.483 *	0.799 *	0.488 *	0.922 **	0.771 *	0.738 *	0.709 *
MBC:MBN	0.221	0.141	0.295	0.218	0.182	0.166	0.172	0.281
MBC:MBP	0.164	0.451 *	0.549 *	0.403 *	0.193	0.154	0.663 *	0.128
MBN:MBP	0.307	0.518 *	0.643 *	0.581 *	0.143	0.167	0.671 *	0.225

*, ** represent significant at *p* < 0.05 and *p* < 0.01. See Figure 2 for abbreviations.
